# Biomarkers of Treatment-Escalation Risk by Obesity Status in Pediatric Asthma: A Development and External Validation Cohort Study

**DOI:** 10.3390/children13091242

**Published:** 2026-09-14

**Authors:** Serdar Göktaş, Mehmet Şirin Kaya

**Affiliations:** 1 Department of Pediatrics, Clinic of Pediatric Immunology and Allergy, Mersin City Training and Research Hospital, 33240 Mersin, Türkiye; 2 Department of Pediatrics, Clinic of Pediatric Immunology and Allergy, Van Training and Research Hospital, 65300 Van, Türkiye; 3 Department of Pediatrics, Clinic of Pediatric Immunology and Allergy, Şanlıurfa Training and Research Hospital, 63200 Şanlıurfa, Türkiye; dr-sirinkaya@hotmail.com

**Keywords:** childhood obesity, CXCL8, external validation, pediatric asthma, prognostic model, TGF-β1, treatment escalation

## Abstract

**Highlights:**

**What are the main findings?**

• TGF-β1 was associated with treatment escalation in obese asthma, whereas CXCL8 was associated with escalation in normal-weight asthma.
• TGF-β1 and CXCL8 models showed improved prognostic performance and retained favorable discrimination in external validation.

**What are the implications of the main findings?**

• Obesity status may be important when interpreting prognostic biomarkers in pediatric asthma.
• Further multicenter validation is required before obesity-stratified biomarker models are used clinically.

**Abstract:**

**Background/Objectives:** Obesity may alter the prognostic value of biomarkers in childhood asthma. We examined whether transforming growth factor beta 1 (TGF-β1), soluble suppression of tumorigenicity 2 (sST2), C-X-C motif chemokine ligand 8 (CXCL8), and insulin-like growth factor 1 (IGF-1) were associated with sustained treatment escalation and whether associations differed by obesity status. **Methods:** The development cohort included 292 children: 90 non-atopic controls, 108 with normal-weight asthma, and 94 with obese asthma; longitudinal analyses included 202 with asthma. Biomarkers were modeled continuously in prespecified Cox models with biomarker-by-obesity interactions, internally validated with 1000 bootstrap resamples, and applied without refitting or recalibration to an independent Van cohort of 180 children with asthma. **Results:** Seventy-five children experienced sustained treatment escalation. TGF-β1 was associated with escalation in obese asthma (hazard ratio [HR] per 500 pg/mL, 2.12; 95% confidence interval [CI], 1.66–2.70; interaction q < 0.001), whereas CXCL8 was associated with escalation in normal-weight asthma (HR per 10 pg/mL, 2.06; 95% CI, 1.67–2.54; interaction q < 0.001). Adding TGF-β1 or CXCL8 increased 12-month time-dependent area under the curve (AUC) from 0.623 to 0.732 and 0.746. In Van, AUCs were 0.751 and 0.744, and interactions remained significant after adjustment (q = 0.036). sST2 showed a weaker obesity-related signal, whereas IGF-1 added little prognostic information. **Conclusions:** Prognostic biomarker associations differed by obesity status. TGF-β1 was associated with escalation in obese asthma, whereas CXCL8 was associated with escalation in normal-weight asthma. Further validation is needed before clinical use.

## 1. Introduction

Asthma is a heterogeneous disease in children. Clinical course, inflammatory patterns, and treatment needs vary across patients [1]. Obesity may add further heterogeneity through metabolic and inflammatory pathways that are not fully captured by routine clinical measures [2].

Several circulating biomarkers are relevant to these pathways. Transforming growth factor beta 1 (TGF-β1) is involved in immune regulation and airway remodeling [3]. Soluble suppression of tumorigenicity 2 (sST2) reflects interleukin-33/ST2 signaling [4]. C-X-C motif chemokine ligand 8 (CXCL8) contributes to neutrophil recruitment and airway inflammation [5]. Insulin-like growth factor 1 (IGF-1) is linked to growth and metabolic signaling [6]. Their prognostic relevance may differ by obesity status.

Most pediatric asthma biomarker studies have focused on cross-sectional differences or current disease activity. Whether baseline biomarker levels are associated with later treatment escalation, whether obesity modifies these associations, and whether biomarkers improve prognostic performance beyond clinical variables remain unclear [7]. External validation is also essential before prognostic models are considered for clinical use.

We evaluated baseline TGF-β1, sST2, CXCL8, and IGF-1 in children with normal-weight asthma, obese asthma, and non-atopic healthy controls. We examined their associations with 12-month sustained treatment escalation, tested biomarker-by-obesity interactions, and assessed incremental prognostic performance. Development models were then evaluated in an independent external cohort.

## 2. Materials and Methods

### 2.1. Study Design and Participants

The study is reported in accordance with the Transparent Reporting of a multivariable prediction model for Individual Prognosis Or Diagnosis (TRIPOD) statement, and the design, analysis, and validation strategy followed established methodological recommendations for prediction model studies [8,9].

This prospective two-center study included a development cohort from Şanlıurfa and an independent external validation cohort from Van. The development cohort was recruited at the Pediatric Immunology and Allergy Clinic at Şanlıurfa Training and Research Hospital (Şanlıurfa, Türkiye) between 1 July and 31 December 2024. Children with asthma were followed for up to 12 months. The 12-month clinical risk period for the development cohort ended by 31 December 2025. Electronic prescription and pharmacy dispensing records used only to adjudicate the 3-month sustainment of late treatment escalations were reviewed through 31 March 2026.

The validation cohort was recruited at the Pediatric Immunology and Allergy Clinic at Van Training and Research Hospital (Van, Türkiye) between 1 October 2024 and 31 March 2025. Children with asthma were followed for up to 12 months. The 12-month clinical risk period for the validation cohort ended by 31 March 2026. Electronic prescription and pharmacy dispensing records used only to adjudicate the 3-month sustainment of late treatment escalations were reviewed through 30 June 2026.

Participants were enrolled consecutively during the prespecified recruitment periods. All consecutive children with asthma who met the eligibility criteria were invited to participate. The same asthma eligibility criteria, clinical assessment procedures, predictor definitions, and primary outcome definition were used at both centers.

Children aged 6–17 years with confirmed asthma were eligible. Asthma was diagnosed by pediatric allergy and immunology specialists according to the 2024 Global Initiative for Asthma (GINA) recommendations [10]. Diagnosis required typical variable respiratory symptoms and documented variable expiratory airflow limitation. Airflow variability was confirmed by bronchodilator responsiveness or previously documented variable airflow limitation. Bronchodilator responsiveness was assessed 10–15 min after 200–400 µg of inhaled salbutamol using age-appropriate GINA 2024 criteria [10].

Obesity was defined as an age- and sex-specific body mass index (BMI) at or above the 95th percentile according to Centers for Disease Control and Prevention growth charts [11]. Normal weight was defined as a BMI from the 5th to below the 85th percentile. Children with a BMI below the 5th percentile or from the 85th to below the 95th percentile were not eligible for the prespecified normal-weight or obese asthma groups. Children receiving biologic therapy at baseline and those with concurrent chronic respiratory disease were excluded.

Atopy was defined as sensitization to at least one common aeroallergen by skin prick testing and/or a serum allergen-specific immunoglobulin E (IgE) level ≥ 0.35 kU/L. A skin prick test was positive when the allergen wheal was at least 3 mm larger than the negative control in the presence of an adequate histamine response.

The development cohort also included non-atopic healthy controls recruited from general pediatric outpatient clinics during routine health visits or pre-procedural evaluations. Controls were age- and sex-frequency-matched to the asthma groups. Healthy controls were required to have an age- and sex-specific BMI from the 5th to below the 85th percentile according to the Centers for Disease Control and Prevention growth charts. They had no history or current symptoms of asthma, allergic rhinitis, atopic dermatitis, food allergy, or chronic respiratory disease. Non-atopic status required negative sensitization testing to the study aeroallergen panel. Serum allergen-specific IgE, when measured, was <0.35 kU/L. Spirometry showed no evidence of airflow obstruction when a technically acceptable and reproducible maneuver was available. Healthy controls were included only in baseline between-group comparisons; longitudinal analyses were restricted to children with asthma.

Participants with a hemolyzed baseline blood sample that precluded biomarker measurement were excluded from the analytic cohort. Children with asthma without usable post-baseline clinical outcome data were also excluded.

### 2.2. Baseline Assessment

Baseline variables included age, sex, BMI percentile, asthma control, GINA treatment step, spirometry, blood eosinophil count, atopy, and serum TGF-β1, sST2, CXCL8, and IGF-1 concentrations. Uncontrolled asthma was defined as an age-appropriate Asthma Control Test (ACT) or Childhood Asthma Control Test (Childhood ACT) score ≤ 19 [12,13].

Spirometry was performed and analyzed according to American Thoracic Society/European Respiratory Society technical standards and was reported when technically acceptable and reproducible maneuvers were available [14]. Forced expiratory volume in 1 s (FEV_1_) percent predicted was calculated using the Global Lung Function Initiative (GLI) 2012 reference equations [15].

Baseline GINA 2024 treatment step was prespecified and modeled as an ordinal numeric covariate, with higher values representing greater treatment intensity. The same coding was retained in the external validation cohort [10].

### 2.3. Biomarker Measurements

Fasting venous blood samples (4–5 mL) were collected between 08:00 and 10:00 after an overnight fast. Samples were allowed to clot for 30 min at room temperature and were centrifuged at 1000× *g* for 15 min at 4 °C. Serum was aliquoted and stored at −80 °C until batch analysis. Repeated freeze–thaw cycles were avoided, and hemolyzed samples were excluded before biomarker measurement.

Serum biomarkers were measured using sandwich enzyme-linked immunosorbent assay kits (Elabscience Biotechnology, Houston, TX, USA) according to the manufacturer’s instructions. TGF-β1 was measured using kit E-EL-H0110 (assay range, 31.25–2000 pg/mL; sensitivity, 18.75 pg/mL). Serum samples underwent the manufacturer-specified acid activation and neutralization procedure before assay. For serum/plasma, this procedure produces a mandatory 1:10 dilution; therefore, TGF-β1 concentrations obtained from the standard curve were multiplied by 10 and are reported throughout the manuscript as serum-equivalent concentrations. No TGF-β1 sample required dilution beyond this mandatory activation dilution because the activated aliquots remained within the assay range. sST2 was measured using kit E-EL-H6082 (assay range, 0.31–20 ng/mL; sensitivity, 0.19 ng/mL). CXCL8 levels were measured using the Elabscience E-EL-H6008 kit (assay range, 7.81–500 pg/mL; sensitivity, 4.69 pg/mL). All sample concentrations fell within the standard curve, and no further dilutions were required. IGF-1 was measured using kit E-EL-H0086 (assay range, 1.56–100 ng/mL; sensitivity, 0.94 ng/mL).

Where additional dilution was required, samples were diluted using the manufacturer-provided sample diluent and reanalyzed, and all reported concentrations were corrected for the applicable dilution factor. For sST2, samples exceeding 20 ng/mL were re-assayed after a 1:5 dilution. In the development cohort, this was required for 86 of 94 (91.5%) samples in the obese-asthma group, 45 of 108 (41.7%) in the normal-weight-asthma group, and 18 of 90 (20.0%) in the non-atopic healthy control group. In the external validation cohort, it was required for 79 of 84 (94.0%) samples in the obese-asthma group and 41 of 96 (42.7%) in the normal-weight-asthma group. A dilution-linearity assessment using three high-concentration serum samples serially diluted 1:2, 1:4, and 1:8 showed recoveries ranging from 94% to 106%. For IGF-1, all samples were analyzed after a 1:10 dilution.

Standards, quality controls, and serum samples were analyzed in duplicate, and concentrations from duplicate measurements were averaged. Intra-assay and inter-assay coefficients of variation were <8% and <10%, respectively. Laboratory personnel were blinded to clinical data, obesity status, and longitudinal outcomes. Both cohorts were analyzed using the same assay platform and laboratory protocol.

### 2.4. Outcome Ascertainment and Follow-Up

The primary outcome was sustained treatment escalation during 12 months of follow-up, defined as the first post-enrollment increase of at least one age-appropriate GINA 2024 treatment step maintained for at least three consecutive months [10].

Participants were evaluated at months 3, 6, 9, and 12 and at unscheduled visits when clinically indicated. Electronic health records and prescription and pharmacy dispensing records were reviewed to document treatment changes. Participants who missed scheduled visits were contacted by telephone to document their current treatment regimen and clinical status.

Short-term treatment changes during acute exacerbations, including systemic corticosteroid courses or step increases lasting less than three months, were not classified as sustained treatment escalation. Potential events were independently reviewed by two pediatric allergy specialists blinded to baseline biomarker concentrations. Disagreements were resolved by consensus with a third investigator.

For time-to-event analyses, the event time was defined as the date on which the qualifying treatment-step increase was initiated, provided that the higher treatment step was subsequently maintained for at least three consecutive months. The 12-month follow-up period defined the time at risk for the initial treatment escalation. For step increases initiated late in follow-up, electronic prescription and pharmacy dispensing records extending beyond the 12-month risk period were reviewed only to determine whether the 3-month sustainment requirement was met. This additional record review did not extend the time at risk. Step increases initiated after the 12-month risk period were not counted as events. Step increases initiated within the risk period but not maintained for three consecutive months were not classified as events.

Participants whose follow-up ended before month 12 without an event were censored at their last clinical contact. Those who completed 12 months without an event were administratively censored at 12 months.

### 2.5. Statistical Analysis

Continuous variables were summarized as mean ± standard deviation (SD) or median with interquartile range (IQR), as appropriate. Categorical variables were summarized as number and percentage. Three-group comparisons used one-way analysis of variance or the Kruskal–Wallis test. Significant overall comparisons were followed by Tukey’s honestly significant difference test or multiplicity-adjusted Dunn pairwise comparisons, as appropriate. Categorical variables were compared using Pearson’s chi-square test. Comparisons restricted to the two asthma groups used Pearson’s chi-square test or the Mann–Whitney U test.

Baseline biomarker concentrations were also summarized by observed sustained treatment escalation within each obesity stratum. Participants censored before month 12 without an event remained in the descriptive “no observed sustained treatment escalation” group but were censored at their observed follow-up time in all time-to-event analyses.

Kaplan–Meier failure estimates were used to display cumulative event probability. Within-stratum TGF-β1 tertiles were plotted for obese asthma and CXCL8 tertiles for normal-weight asthma. Tertiles were compared using global log-rank tests and were used only for visualization. Biomarkers were modeled continuously in regression analyses.

Each biomarker was evaluated in a separate prespecified Cox proportional hazards model. Models included the continuous biomarker, obesity status, the biomarker-by-obesity interaction, age, sex, baseline FEV_1_ percent predicted, uncontrolled asthma, and baseline GINA treatment step. Before model fitting, each biomarker was centered at its development-cohort median and scaled in the unit used for the hazard ratio: ZTGF = (TGF-β1 − 1500)/500, ZsST2 = (sST2 − 18)/10, ZCXCL8 = (CXCL8 − 40)/10, and ZIGF-1 = (IGF-1 − 230)/50. The biomarker-centering values were prespecified as the medians of the full development cohort (n = 292), including healthy controls; this linear translation does not alter stratum-specific biomarker hazard ratios, biomarker-by-obesity interactions, or model performance. The centered and scaled biomarker variable was used both as the biomarker main effect and to construct the biomarker-by-obesity interaction term. Hazard ratios (HRs) were therefore expressed per 500 pg/mL increase in TGF-β1, 10 ng/mL increase in sST2, 10 pg/mL increase in CXCL8, and 50 ng/mL increase in IGF-1. The full predictor coding and model equations are provided in Supplementary Table S1.

Stratum-specific HRs were derived from the same interaction model. The biomarker main effect represented the association in normal-weight asthma, and the obese-asthma estimate incorporated the biomarker main effect and interaction term. Interaction *p* values tested effect modification by obesity status. Multiplicity across the four prespecified interaction tests was controlled using the Benjamini–Hochberg (BH) procedure [16].

Missing baseline FEV_1_ values were handled using multiple imputation by chained equations in the primary analyses. Twenty imputed datasets were generated. The imputation model included the variables used in the Cox models, the event indicator, and the Nelson–Aalen estimator of the cumulative hazard. Cox regression coefficients were pooled using Rubin’s rules. Obesity-stratum mean imputation and complete-case analyses were performed as sensitivity analyses.

The proportional hazards assumption was assessed using Schoenfeld residuals for individual covariates and globally [17]. Functional forms of continuous predictors were assessed using Martingale residuals. Ridge-penalized Cox regression was performed as a sensitivity analysis.

Analyses were performed using IBM SPSS Statistics version 26.0 and R version 4.3.1.

### 2.6. Internal Validation and Model Performance

The clinical model included age, sex, obesity status, baseline FEV_1_ percent predicted, uncontrolled asthma, and baseline GINA treatment step. Each biomarker model added one continuous biomarker and its biomarker-by-obesity interaction.

Internal validation used 1000 bootstrap resamples with optimism correction [18]. Overall discrimination was assessed using Uno’s C-index [19]. Twelve-month discrimination was assessed using the cumulative/dynamic time-dependent area under the receiver operating characteristic curve (AUC) with inverse probability of censoring weighting [20]. Prediction error was assessed with the 12-month Brier score, and calibration with the calibration intercept and slope. ΔC-index represented the optimism-corrected difference from the clinical model and was calculated from unrounded estimates. Performance measures were calculated in each of the 20 imputed datasets and then averaged across datasets. Twelve-month calibration curves were constructed using local Kaplan–Meier estimates to account for censoring.

As a secondary sensitivity analysis of incremental model fit, each biomarker model was compared with the clinical model using a two-degree-of-freedom likelihood-ratio test jointly evaluating the biomarker main effect and biomarker-by-obesity interaction in the obesity-stratum mean-imputed dataset used in the original analysis. The four comparisons were adjusted using the Benjamini–Hochberg procedure. These likelihood-ratio tests were not pooled across the multiply imputed datasets and are therefore reported as sensitivity analyses. Primary coefficient estimation and model-performance assessment used the multiple-imputation framework described above.

Discrimination, prediction error, and calibration estimates were corrected for optimism. Optimism correction was applied pointwise to the development time-dependent receiver operating characteristic (ROC) curves.

### 2.7. External Validation Procedures

Development-model regression coefficients, predictor coding, scaling constants, biomarker-centering values, and model-specific 12-month baseline cumulative hazards were fixed and applied to the Van cohort without refitting or recalibration. The full clinical and biomarker-specific model specifications are provided in Supplementary Table S1.

Missing baseline FEV_1_ values in the validation cohort were handled using multiple imputation by chained equations. The frozen development-model parameters were applied without refitting or recalibration. Performance measures were calculated in each imputed dataset and averaged across the 20 datasets.

External performance was assessed using Uno’s C-index, ΔC-index, the 12-month cumulative/dynamic time-dependent AUC, the 12-month Brier score, calibration intercept, and calibration slope. Bootstrap 95% confidence intervals (CIs) were obtained by resampling the validation cohort while holding the development-model parameters fixed. No optimism correction was applied.

Each biomarker-specific Cox model was also re-estimated in the Van cohort using the same covariate specification as in development. Biomarker-by-obesity interaction *p* values were adjusted across the four biomarkers using the BH procedure.

### 2.8. Study Size

All consecutive eligible participants presenting during the prespecified 6-month recruitment periods were invited to participate. No separate sample-size target was used to restrict enrollment. The cohorts therefore comprised all eligible participants enrolled during their respective recruitment periods.

## 3. Results

### 3.1. Study Population and Baseline Characteristics

**Figure 1:**
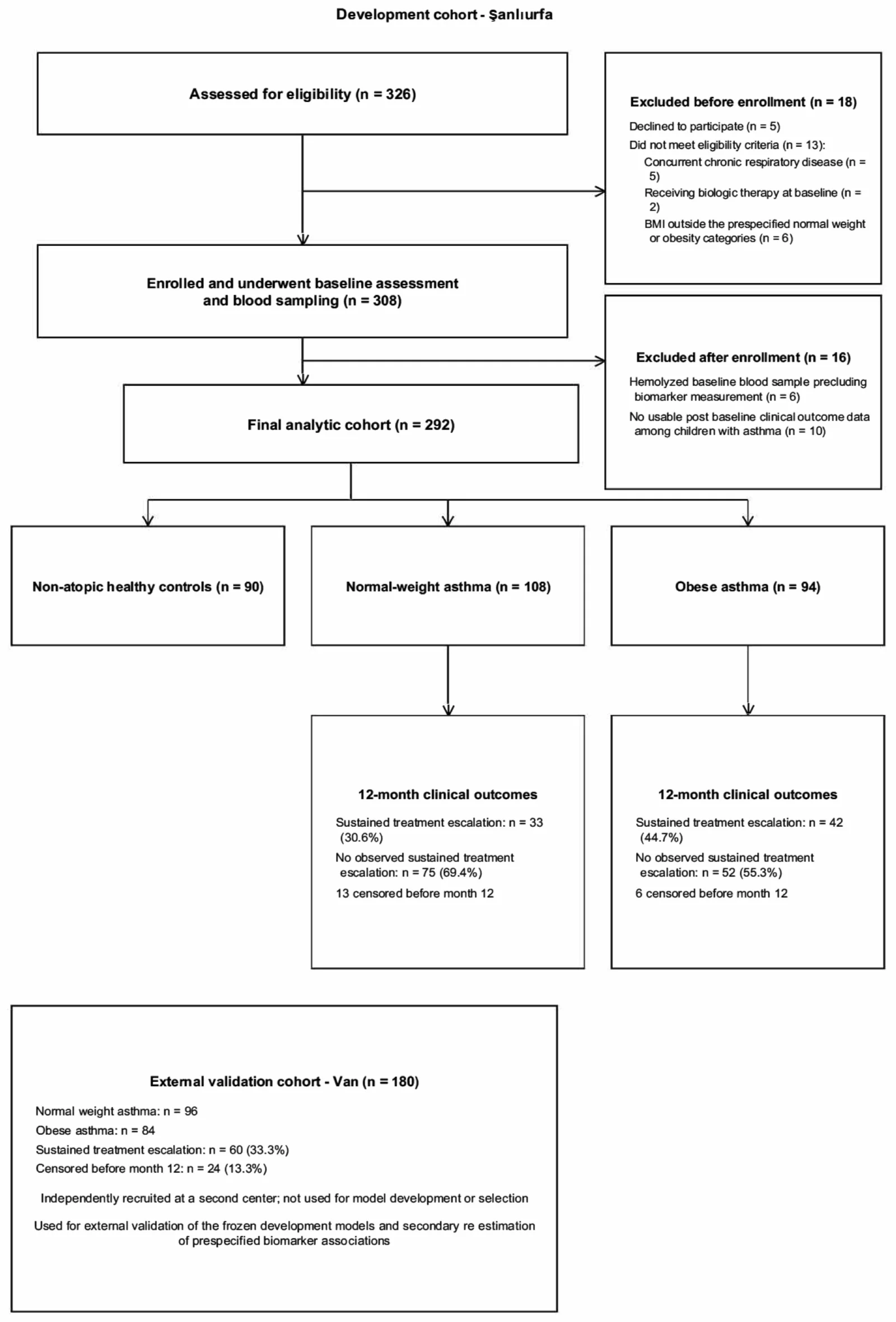
Study flow diagram. Participant screening, exclusions, study-group classification, follow-up, and the external validation cohort are shown. Arrows indicate participant flow through screening, enrollment, exclusions, study-group allocation, follow-up, and external validation. Non-atopic healthy controls were included only in baseline comparisons. Sustained treatment escalation was defined as an increase of at least one GINA treatment step maintained for at least three consecutive months. Children without an event who ended follow-up before 12 months were censored at their last clinical contact. The Van cohort was used for external validation of the development models. Abbreviations: BMI, body mass index; GINA, Global Initiative for Asthma.

Of 326 children assessed for eligibility in the development cohort, 18 were excluded before enrollment: five declined participation, five had concurrent chronic respiratory disease, two were receiving biologic therapy at baseline, and six had a BMI outside the prespecified normal-weight or obesity categories. Of 308 enrolled children, 16 were subsequently excluded because of a hemolyzed baseline blood sample (n = 6) or unavailable post-baseline clinical outcome data among children with asthma (n = 10). The final analytic cohort included 292 participants: 90 non-atopic healthy controls, 108 children with normal-weight asthma, and 94 children with obese asthma (Figure 1). Longitudinal analyses included the 202 children with asthma.

Baseline demographic and clinical characteristics are presented in Table 1. Uncontrolled asthma was more frequent in obese asthma than in normal-weight asthma. TGF-β1 and sST2 concentrations were higher in obese asthma than in both normal-weight asthma and non-atopic healthy controls. CXCL8 concentrations were higher in both asthma groups than in controls but did not differ between the two asthma groups. No Tukey-adjusted pairwise comparison for IGF-1 reached statistical significance.

### 3.2. Biomarkers and Sustained Treatment Escalation

Sustained treatment escalation occurred in 33 children with normal-weight asthma and 42 with obese asthma. Thirteen normal-weight and six obese children were censored before month 12 (Figure 1).

Baseline biomarker concentrations according to observed treatment escalation are shown in Table 2. In obese asthma, TGF-β1 and sST2 concentrations were higher among children with sustained treatment escalation. In normal-weight asthma, CXCL8 concentrations were higher among children with sustained treatment escalation. The no-observed-event groups included participants censored before 12 months and were not treated as confirmed event-free observations in time-to-event analyses.

**Figure 2:**
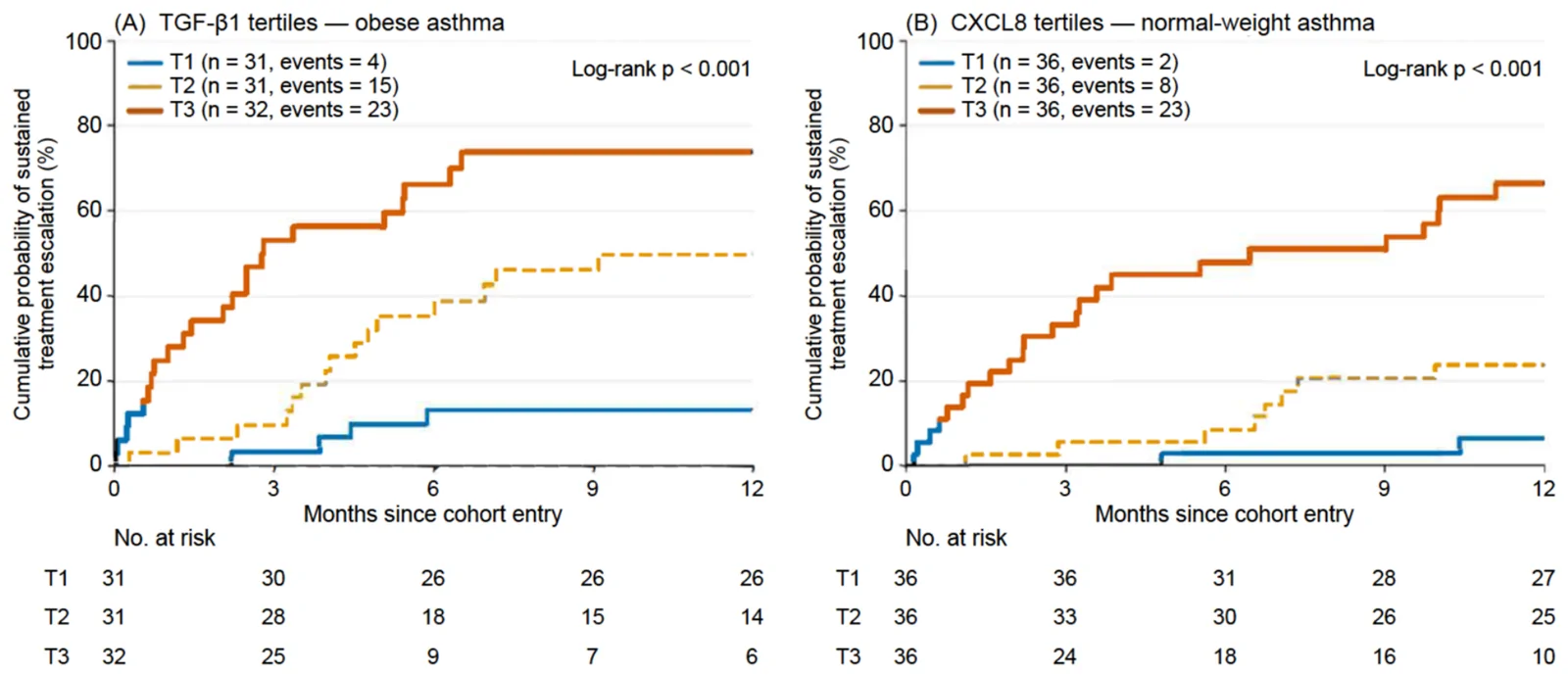
Cumulative probability of sustained treatment escalation according to baseline biomarker tertiles within obesity strata. Kaplan–Meier failure estimates are shown for TGF-β1 tertiles in obese asthma (**A**) and CXCL8 tertiles in normal-weight asthma (**B**). T1, T2, and T3 denote the lowest, middle, and highest within-stratum tertiles. *p* values are from global log-rank tests. Tertiles were used only for visualization; biomarkers were modeled continuously in the Cox regression analyses. Number-at-risk tables account for events and censoring during follow-up. Abbreviations: CXCL8, C-X-C motif chemokine ligand 8; T1, lowest tertile; T2, middle tertile; T3, highest tertile; TGF-β1, transforming growth factor beta 1.

Kaplan–Meier failure curves are shown in Figure 2. Event probability increased across TGF-β1 tertiles in obese asthma and across CXCL8 tertiles in normal-weight asthma (both global log-rank *p* < 0.001).

### 3.3. Adjusted Biomarker Associations

Adjusted Cox regression results are presented in Table 3. For TGF-β1, the biomarker-by-obesity interaction was significant (*p* < 0.001; BH-adjusted q < 0.001). Each 500 pg/mL increase was associated with an HR of 2.12 (95% CI, 1.66–2.70) in obese asthma and 0.90 (95% CI, 0.63–1.30) in normal-weight asthma. For sST2, each 10 ng/mL increase was associated with an HR of 1.50 (95% CI, 1.14–1.98) in obese asthma and 0.70 (95% CI, 0.37–1.32) in normal-weight asthma (interaction *p* = 0.024; q = 0.033).

For CXCL8, each 10 pg/mL increase was associated with an HR of 2.06 (95% CI, 1.67–2.54) in normal-weight asthma and 0.92 (95% CI, 0.78–1.10) in obese asthma (interaction *p* < 0.001; q < 0.001). The IGF-1 interaction was not significant (*p* = 0.920; q = 0.920).

No violation of the proportional hazards assumption was detected in covariate-specific or global Schoenfeld residual tests (all *p* > 0.10). Inspection of Martingale residuals did not indicate important departures from linearity for the continuous predictors. Ridge-penalized Cox models produced biomarker-by-obesity interaction estimates similar to those from the unpenalized models. Multiple-imputation analyses produced estimates similar to the complete-case analyses. The direction of the biomarker-by-obesity interactions was unchanged in the mean-imputation and complete-case sensitivity analyses.

### 3.4. Internal Validation

**Figure 3:**
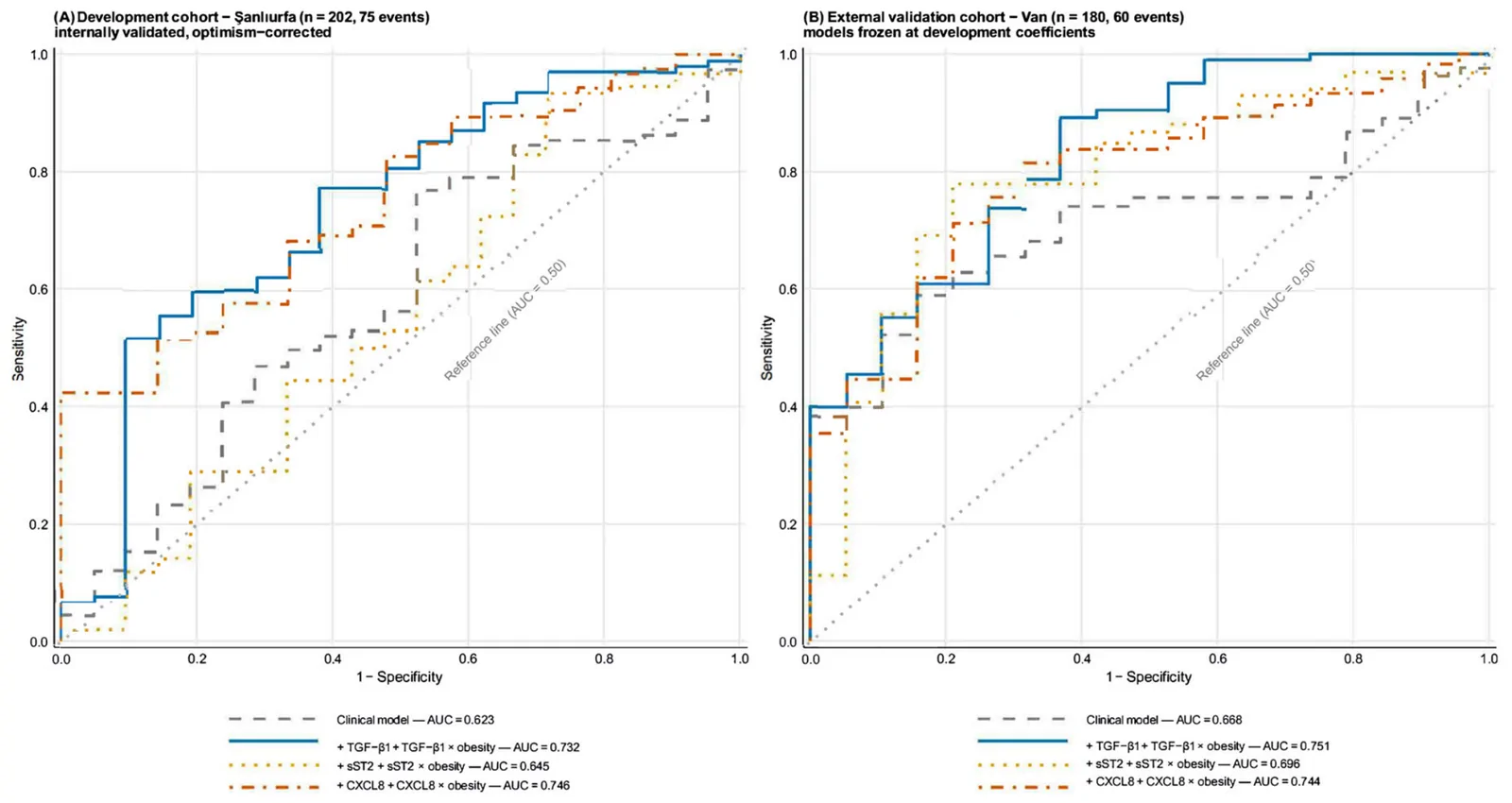
Time-dependent receiver operating characteristic curves for sustained treatment escalation at 12 months. Curves are shown for the development cohort (**A**) and external validation cohort (**B**). Development-cohort curves were derived from the 1000-resample bootstrap analysis with optimism correction. In the external validation cohort, the frozen development models were applied without refitting or recalibration. The clinical model included age, sex, obesity status, baseline FEV_1_ percent predicted, uncontrolled asthma, and baseline GINA treatment step. Each biomarker model added the corresponding biomarker and its biomarker-by-obesity interaction. Time-dependent AUCs were estimated with inverse probability of censoring weighting. Figure 3 displays the clinical, TGF-β1, sST2, and CXCL8 models; IGF-1 results are reported in Table 4. The diagonal line represents an AUC of 0.50. Abbreviations: AUC, area under the receiver operating characteristic curve; CXCL8, C-X-C motif chemokine ligand 8; FEV_1_, forced expiratory volume in 1 s; GINA, Global Initiative for Asthma; IGF-1, insulin-like growth factor 1; sST2, soluble suppression of tumorigenicity 2; TGF-β1, transforming growth factor beta 1.

Internal validation results are presented in Table 4. Adding TGF-β1 and its obesity interaction improved discrimination and reduced the 12-month Brier score compared with the clinical model. The CXCL8 model showed a similar improvement. The sST2 model produced little change in discrimination; a secondary mean-imputation sensitivity likelihood-ratio test suggested incremental model fit. IGF-1 did not improve prognostic performance (Table 4, Figure 3A).

### 3.5. External Validation

The external validation cohort included 180 children with asthma, of whom 60 experienced sustained treatment escalation (Table 5A). When the frozen development models were applied without refitting or recalibration, the TGF-β1 and CXCL8 models showed higher discrimination and lower prediction error than the clinical model. The sST2 model showed a smaller improvement, whereas IGF-1 added little prognostic information (Table 5B, Figure 3B). In Cox models re-estimated in the Van cohort, the biomarker-by-obesity interactions remained significant after multiplicity adjustment for TGF-β1 and CXCL8 but not for sST2 or IGF-1 (Table 5C). Twelve-month calibration curves are shown in Supplementary Figure S1B.

## 4. Discussion

The prognostic associations of circulating biomarkers differed by obesity status in children with asthma. TGF-β1 was associated with treatment escalation in obese asthma, whereas CXCL8 was associated with treatment escalation in normal-weight asthma. The TGF-β1 and CXCL8 models also showed higher discrimination than the clinical model in the external cohort. sST2 provided a smaller increment in prognostic performance, while IGF-1 added little prognostic information.

Prognostic biomarker research in pediatric asthma has focused largely on type 2 inflammation. Blood eosinophil count and fractional exhaled nitric oxide have been associated with asthma attacks and treatment response [21,22]. Total and specific IgE help characterize the type 2-high phenotype in children [23]. Periostin has also been studied as a type 2 marker, although interpretation of serum levels in children is affected by bone growth and turnover [24]. Our study examined four other circulating biomarkers and evaluated whether their prognostic associations differed by obesity status.

Previous work on TGF-β1 in asthma has been mainly cross-sectional or mechanistic. TGF-β1 is involved in airway remodeling, although associations with asthma severity have varied across studies [3]. Serum TGF-β1 has been reported to be higher in children with atopic asthma without a clear relationship with lung function [25]. Higher circulating TGF-β1 has also been reported in children with obesity [26]. In our study, baseline TGF-β1 was associated with subsequent treatment escalation in obese asthma but not in normal-weight asthma. The biomarker-by-obesity interaction was also observed in the validation cohort.

CXCL8 has been linked to neutrophilic airway inflammation and asthma severity [5]. Obesity has also been associated with a more neutrophilic inflammatory pattern in asthma [27]. In our cohort, baseline CXCL8 concentrations did not differ between normal-weight and obese asthma. However, CXCL8 was associated with treatment escalation in normal-weight asthma and not in obese asthma. These findings show that cross-sectional biomarker concentrations and longitudinal prognostic associations may differ.

The IL-33/ST2 pathway has been linked to asthma inflammation and exacerbation [4]. Serum sST2 has been associated with severe exacerbation and neutrophilic inflammation in adults [28], and pediatric studies have also evaluated sST2 as a marker of asthma severity [29]. In our study, the sST2-by-obesity interaction was present in the development cohort but was not significant after multiplicity adjustment in the validation cohort. Further studies are needed to assess its prognostic value.

Evidence for IGF-1 in asthma is more limited. IGF-1 signaling has been implicated in airway remodeling, and human studies have reported associations with asthma and lung function [30]. In children, circulating IGF-1 is also related to growth and metabolic factors [6,31]. In our study, IGF-1 showed no consistent association with treatment escalation and did not improve prognostic performance.

Previous studies have shown that obesity can influence asthma phenotype, treatment response, and exacerbation risk in children [2,32,33,34]. Our findings add to this literature by showing that the prognostic associations of TGF-β1 and CXCL8 differed by obesity status. These results support evaluation of biomarker-by-obesity interactions in future prognostic studies of pediatric asthma.

The study has several strengths. Biomarkers were modeled continuously in prespecified interaction models rather than converted into data-derived clinical cutoffs, which avoids loss of information associated with dichotomization [35]. Time-to-event methods accounted for incomplete follow-up. Prognostic performance was assessed using discrimination, calibration, and prediction error. Internal validation used bootstrap optimism correction, and the development models were evaluated in an independent cohort without refitting or recalibration.

Several limitations should be considered. The observational design does not establish causal effects of the biomarkers. Serum concentrations may not fully reflect airway-level biological activity, and biomarkers were measured only at baseline. Treatment escalation is a clinician-driven outcome and may be influenced by assessment and prescribing practice, so confounding by indication cannot be excluded [36]. With 75 events and eight model parameters, the interaction estimates require confirmation in larger cohorts. The incremental value of the candidate biomarkers was evaluated against a prespecified clinical model rather than against a model incorporating established type 2 biomarkers such as blood eosinophils or FeNO; comparative biomarker models should be evaluated in larger cohorts. Children with BMI from the 85th to below the 95th percentile were excluded by design, so the findings should not be extrapolated to the overweight range. The cohorts also differed in case mix: children in the Van cohort were younger on average and had somewhat lower lung function and greater baseline treatment intensity, particularly in the obese-asthma group. Because baseline GINA treatment step in the development cohort ranged from Steps 1 to 3, application of the frozen ordinal treatment-step coefficient to participants at Step 4 in the external validation cohort involved limited extrapolation and should be interpreted cautiously. Missing baseline FEV_1_ values were limited to seven children in each asthma cohort and were handled using multiple imputation in the primary analyses; mean-imputation and complete-case sensitivity analyses produced similar results. Finally, both cohorts were recruited from two centers in Türkiye. Validation in broader populations and different clinical settings is needed before these biomarkers are used for individual risk assessment.

## 5. Conclusions

Biomarker associations with sustained treatment escalation differed by obesity status. TGF-β1 was associated with treatment escalation in obese asthma, whereas CXCL8 was associated with treatment escalation in normal-weight asthma. sST2 provided more limited prognostic information, and IGF-1 added little. Further validation is needed before obesity-stratified biomarker models are used in clinical practice.

## Figures and Tables

**Table 1:** Baseline demographic, clinical, lung function, and biomarker characteristics of the study groups in the development cohort (Şanlıurfa).

Parameter	Non-Atopic Healthy Controls (n = 90)	Normal-Weight Asthma (n = 108)	Obese Asthma (n = 94)	*p* Value
**Demographic characteristics**				
Age, years, mean ± SD	11.5 ± 2.5	11.2 ± 2.1	11.5 ± 2.5	0.513 ^a^
Male sex, n (%)	42 (46.7)	60 (55.6)	51 (54.3)	0.417 ^b^
BMI percentile, median (IQR)	47.5 (38.4–62.0)	56.6 (45.8–67.0)	97.4 (96.4–98.4)	<0.001 ^c^
**Clinical and lung function characteristics**				
Uncontrolled asthma, defined by an age-appropriate ACT or Childhood ACT score ≤ 19, n (%)	NA	47 (43.5)	54 (57.4)	0.048 ^d^
Baseline GINA 2024 treatment step, median (IQR)	NA	2 (2–2)	2 (2–3)	0.485 ^e^
Step 1/Step 2/Step 3, n	NA	20/62/26	21/41/32	—
FEV_1_, % predicted, mean ± SD	105.8 ± 8.0	91.4 ± 11.7	87.7 ± 11.6	<0.001 ^a,f^
FEV_1_/FVC, mean ± SD	0.88 ± 0.04	0.82 ± 0.06	0.79 ± 0.06	<0.001 ^a,f^
Blood eosinophil count, cells/µL, median (IQR)	126 (91–164)	358 (244–450)	337 (256–479)	<0.001 ^c^
Atopy, n (%)	0 (0.0)	72 (66.7)	59 (62.8)	0.563 ^d^
**Baseline serum biomarkers**				
TGF-β1, pg/mL, mean ± SD	1371 ± 377	1581 ± 459	2915 ± 746	<0.001 ^a^
sST2, ng/mL, mean ± SD	16.8 ± 5.2	19.3 ± 5.9	39.8 ± 14.1	<0.001 ^a^
CXCL8, pg/mL, mean ± SD	14.3 ± 3.9	44.8 ± 16.9	44.2 ± 19.7	<0.001 ^a^
IGF-1, ng/mL, mean ± SD	222.4 ± 58.5	226.1 ± 68.8	245.2 ± 70.2	0.042 ^a^

Data are presented as mean ± SD, median (IQR), or number (percentage), as appropriate. NA indicates not applicable. ^a^ One-way analysis of variance with Tukey-adjusted pairwise comparisons when the overall test was significant. Post hoc testing showed higher TGF-β1 and sST2 in obese asthma than in both other groups, and higher CXCL8 in both asthma groups than in controls. No Tukey-adjusted pairwise comparison for IGF-1 was significant. ^b^ Pearson’s chi-square test across the three groups. ^c^ Kruskal–Wallis test with multiplicity-adjusted Dunn pairwise comparisons when the overall test was significant. ^d^ Pearson’s chi-square test comparing the two asthma groups. ^e^ Mann–Whitney U test comparing the two asthma groups. ^f^ Spirometry results are based on technically acceptable and reproducible measurements from 89 controls, 105 children with normal-weight asthma, and 90 children with obese asthma. FEV_1_ percent predicted was calculated using GLI 2012 reference equations [15]. Abbreviations: ACT, Asthma Control Test; BMI, body mass index; CXCL8, C-X-C motif chemokine ligand 8; FEV_1_, forced expiratory volume in 1 s; FVC, forced vital capacity; GINA, Global Initiative for Asthma; GLI, Global Lung Funtion Initiative; IGF-1, insulin-like growth factor 1; IQR, interquartile range; NA, not applicable; SD, standard deviation; sST2, soluble suppression of tumorigenicity 2; TGF-β1, transforming growth factor beta 1.

**Table 2:** Baseline serum biomarker concentrations according to observed sustained treatment escalation during follow-up, stratified by obesity status.

Parameter	Normal-Weight Asthma: No Observed Sustained Treatment Escalation (n = 75)	Normal-Weight Asthma: Sustained Treatment Escalation (n = 33)	Obese Asthma: No Observed Sustained Treatment Escalation (n = 52)	Obese Asthma: Sustained Treatment Escalation (n = 42)
TGF-β1, pg/mL, mean ± SD	1579 ± 486	1585 ± 397	2566 ± 616	3348 ± 666
sST2, ng/mL, mean ± SD	19.5 ± 5.9	18.8 ± 5.8	34.7 ± 12.7	46.1 ± 13.4
CXCL8, pg/mL, mean ± SD	38.6 ± 13.7	58.9 ± 15.1	45.9 ± 19.6	42.2 ± 19.9
IGF-1, ng/mL, mean ± SD	222.2 ± 68.7	234.9 ± 69.4	239.0 ± 68.6	252.8 ± 72.3

Data are presented as mean ± SD. Sustained treatment escalation was defined as an increase of at least one GINA treatment step maintained for at least three consecutive months. The “no observed sustained treatment escalation” groups include participants with no recorded event during their available follow-up, including 13 children with normal-weight asthma and 6 with obese asthma who were censored before 12 months. These participants were censored at their last clinical contact in all time-to-event analyses. Biomarker results were unavailable to treating clinicians and outcome adjudicators during follow-up. Abbreviations: CXCL8, C-X-C motif chemokine ligand 8; GINA, Global Initiative for Asthma; IGF-1, insulin-like growth factor 1; SD, standard deviation; sST2, soluble suppression of tumorigenicity 2; TGF-β1, transforming growth factor beta 1.

**Table 3:** Adjusted associations of baseline serum biomarkers with time to sustained treatment escalation and effect modification by obesity status.

Biomarker and Modeled Increment	Adjusted HR in Normal-Weight Asthma (95% CI)	Adjusted HR in Obese Asthma (95% CI)	*p* for Interaction	BH-Adjusted q Value
TGF-β1, per 500-pg/mL increase	0.90 (0.63–1.30)	2.12 (1.66–2.70)	<0.001	<0.001
sST2, per 10-ng/mL increase	0.70 (0.37–1.32)	1.50 (1.14–1.98)	0.024	0.033
CXCL8, per 10-pg/mL increase	2.06 (1.67–2.54)	0.92 (0.78–1.10)	<0.001	<0.001
IGF-1, per 50-ng/mL increase	1.14 (0.88–1.47)	1.12 (0.90–1.39)	0.920	0.920

Data are presented as adjusted HRs with 95% CIs. Each biomarker was evaluated in a separate Cox model in the development asthma cohort (n = 202; 75 events). Models included the biomarker, obesity status, the biomarker-by-obesity interaction, age, sex, baseline FEV_1_ percent predicted, uncontrolled asthma, and baseline GINA treatment step. Missing baseline FEV_1_ values were handled using multiple imputation. Participants without an event were censored at their last clinical contact or at 12 months. Stratum-specific HRs were derived from the same interaction model. *p* values test the biomarker-by-obesity interaction, and q values are BH-adjusted across the four prespecified interaction tests. Abbreviations: ACT, Asthma Control Test; BH, Benjamini–Hochberg; CI, confidence interval; CXCL8, C-X-C motif chemokine ligand 8; FEV_1_, forced expiratory volume in 1 s; GINA, Global Initiative for Asthma; HR, hazard ratio; IGF-1, insulin-like growth factor 1; sST2, soluble suppression of tumorigenicity 2; TGF-β1, transforming growth factor beta 1.

**Table 4:** Internally validated incremental prognostic performance of baseline serum biomarkers for sustained treatment escalation over 12 months in the development cohort (Şanlıurfa). (**A**) Discrimination and prediction error. (**B**) Calibration and sensitivity analysis of incremental model fit.

**(A)**				
**Model**	**Optimism-Corrected Uno’s C-Index (95% CI)**	**ΔC-Index vs. Clinical Model (95% CI)**	**12-Month Time-Dependent AUC (95% CI)**	**12-Month Brier Score (95% CI)**
Clinical model	0.617 (0.560–0.671)	Reference	0.623 (0.550–0.686)	0.235 (0.214–0.266)
Clinical model + TGF-β1 + TGF-β1 × obesity	0.701 (0.644–0.758)	+0.085 (0.033–0.128)	0.732 (0.664–0.796)	0.205 (0.176–0.239)
Clinical model + sST2 + sST2 × obesity	0.630 (0.572–0.684)	+0.013 (−0.037 to 0.041)	0.645 (0.573–0.709)	0.227 (0.204–0.260)
Clinical model + CXCL8 + CXCL8 × obesity	0.717 (0.668–0.769)	+0.100 (0.045–0.144)	0.746 (0.685–0.808)	0.201 (0.175–0.234)
Clinical model + IGF-1 + IGF-1 × obesity	0.609 (0.552–0.663)	−0.008 (−0.040 to 0.010)	0.618 (0.547–0.679)	0.238 (0.214–0.270)
**(B)**				
**Model**	**12-Month Calibration Intercept**	**Calibration Slope**	**BH-Adjusted Likelihood-Ratio q Value (Mean-Imputation Sensitivity Analysis)**	
Clinical model	−0.01	0.79	Reference	
Clinical model + TGF-β1 + TGF-β1 × obesity	0.00	0.86	<0.001	
Clinical model + sST2 + sST2 × obesity	0.00	0.81	0.006	
Clinical model + CXCL8 + CXCL8 × obesity	0.01	0.86	<0.001	
Clinical model + IGF-1 + IGF-1 × obesity	−0.01	0.73	0.377	

Data are presented as optimism-corrected performance estimates. The development asthma cohort included 202 children and 75 sustained treatment-escalation events. The clinical model included age, sex, obesity status, baseline FEV_1_ percent predicted, uncontrolled asthma, and baseline GINA treatment step. Each biomarker model added one continuous biomarker and its biomarker-by-obesity interaction. Missing baseline FEV_1_ values were handled using multiple imputation in the primary analyses. Participants without an event were censored at their last clinical contact or at 12 months. Internal validation used 1000 bootstrap resamples with optimism correction. Uno’s C-index assessed overall discrimination, the 12-month time-dependent AUC assessed discrimination at 12 months, and the Brier score assessed prediction error. ΔC-index is the difference from the clinical model. The BH-adjusted likelihood-ratio q values were obtained from the obesity-stratum mean-imputed dataset as a secondary sensitivity analysis and jointly test the biomarker main effect and biomarker-by-obesity interaction; these likelihood-ratio tests were not pooled across the multiply imputed datasets. Calibration intercepts and slopes are presented as optimism-corrected point estimates, and calibration across the predicted-risk range is shown in Supplementary Figure S1. Abbreviations: ACT, Asthma Control Test; AUC, area under the receiver operating characteristic curve; BH, Benjamini–Hochberg; CI, confidence interval; CXCL8, C-X-C motif chemokine ligand 8; FEV_1_, forced expiratory volume in 1 s; GINA, Global Initiative for Asthma; IGF-1, insulin-like growth factor 1; sST2, soluble suppression of tumorigenicity 2; TGF-β1, transforming growth factor beta 1.

**Table 5:** External validation of the development models in the independent Van cohort. (**A**) Validation cohort characteristics and follow-up outcomes. (**B**) Performance of frozen development models in the Van cohort. (**C**) Stratum-specific associations from Cox models re-estimated in the Van cohort.

**(A)**						
**Parameter**		**Normal-Weight Asthma (n = 96)**		**Obese Asthma (n = 84)**		**All (N = 180)**
Age, years, mean ± SD		9.2 ± 2.4		9.8 ± 2.6		9.5 ± 2.5
Male sex, n (%)		54 (56.2)		46 (54.8)		100 (55.6)
Baseline FEV_1_, % predicted, mean ± SD		86.4 ± 12.1		83.2 ± 11.5		84.9 ± 11.9
Uncontrolled asthma, n (%)		35 (36.5)		42 (50.0)		77 (42.8)
Baseline GINA 2024 treatment step [10], median (IQR)		2 (2–3)		3 (2–4)		2 (2–3)
Missing baseline FEV_1_, n (%)		4 (4.2)		3 (3.6)		7 (3.9)
TGF-β1, pg/mL, mean ± SD		1616 ± 522		2814 ± 854		2175 ± 918
sST2, ng/mL, mean ± SD		19.6 ± 6.4		43.3 ± 13.3		30.7 ± 15.6
CXCL8, pg/mL, mean ± SD		42.7 ± 19.0		40.7 ± 16.6		41.8 ± 17.9
IGF-1, ng/mL, mean ± SD		237.5 ± 71.9		242.8 ± 67.5		240.0 ± 69.7
Sustained treatment escalation, n (%)		26 (27.1)		34 (40.5)		60 (33.3)
Early censoring before month 12, n (%)		13 (13.5)		11 (13.1)		24 (13.3)
Event-free completion of 12-month follow-up, n (%)		57 (59.4)		39 (46.4)		96 (53.3)
**(B)**						
**Frozen Model**	**Uno’s C-Index (95% CI)**	**ΔC-Index vs. Clinical Model (95% CI)**	**12-Month Time-Dependent AUC (95% CI)**	**12-Month Brier Score (95% CI)**	**Calibration Intercept (95% CI)**	**Calibration Slope (95% CI)**
Clinical model	0.655 (0.592–0.718)	Reference	0.668 (0.601–0.735)	0.205 (0.175–0.235)	0.12 (−0.08 to 0.32)	0.88 (0.75–1.01)
Clinical model + TGF-β1 + TGF-β1 × obesity	0.738 (0.675–0.801)	+0.083 (0.031–0.135)	0.751 (0.685–0.817)	0.170 (0.142–0.198)	0.05 (−0.14 to 0.24)	0.94 (0.82–1.06)
Clinical model + sST2 + sST2 × obesity	0.684 (0.615–0.753)	+0.029 (−0.005 to 0.063)	0.696 (0.628–0.764)	0.192 (0.162–0.222)	0.09 (−0.10 to 0.28)	0.90 (0.78–1.03)
Clinical model + CXCL8 + CXCL8 × obesity	0.732 (0.670–0.794)	+0.077 (0.025–0.129)	0.744 (0.678–0.810)	0.174 (0.146–0.202)	0.07 (−0.11 to 0.25)	0.93 (0.80–1.05)
Clinical model + IGF-1 + IGF-1 × obesity	0.658 (0.595–0.721)	+0.003 (−0.025 to 0.031)	0.671 (0.605–0.737)	0.202 (0.172–0.232)	0.11 (−0.09 to 0.31)	0.89 (0.76–1.02)
**(C)**						
**Biomarker and Modeled Increment**	**Adjusted HR in Normal-Weight Asthma (95% CI)**		**Adjusted HR in Obese Asthma (95% CI)**		***p*** **for Interaction**	**BH-Adjusted q Value**
TGF-β1, per 500-pg/mL increase	1.12 (0.75–1.68)		2.38 (1.45–3.90)		0.012	0.036
sST2, per 10-ng/mL increase	1.10 (0.70–1.72)		1.65 (1.05–2.58)		0.085	0.113
CXCL8, per 10-pg/mL increase	2.25 (1.38–3.66)		1.18 (0.80–1.74)		0.018	0.036
IGF-1, per 50-ng/mL increase	1.06 (0.78–1.44)		1.11 (0.82–1.50)		0.760	0.760

Data are presented as mean ± SD, median (IQR), number (percentage), performance estimates with 95% CIs, or adjusted HRs with 95% CIs, as appropriate. Baseline FEV_1_ summaries in Panel A are based on 173 children with available spirometry. Participants without an event were censored at their last clinical contact or at 12 months. Missing baseline FEV_1_ values used in the model analyses were handled using multiple imputation. In Panel B, the clinical model included age, sex, obesity status, baseline FEV_1_ percent predicted, uncontrolled asthma, and baseline GINA treatment step. Each biomarker model added the corresponding biomarker and its biomarker-by-obesity interaction. Development-model coefficients and baseline cumulative hazards were applied without refitting or recalibration. Uno’s C-index assessed overall discrimination, the time-dependent AUC assessed discrimination at 12 months, and the Brier score assessed prediction error. No optimism correction was applied. In Panel C, each biomarker-specific Cox model was re-estimated using the same covariates as in the development cohort. *p* values test the biomarker-by-obesity interaction, and q values are BH-adjusted across the four prespecified interaction tests. Abbreviations: ACT, Asthma Control Test; AUC, area under the receiver operating characteristic curve; BH, Benjamini–Hochberg; CI, confidence interval; CXCL8, C-X-C motif chemokine ligand 8; FEV_1_, forced expiratory volume in 1 s; GINA, Global Initiative for Asthma; HR, hazard ratio; IGF-1, insulin-like growth factor 1; IQR, interquartile range; SD, standard deviation; sST2, soluble suppression of tumorigenicity 2; TGF-β1, transforming growth factor beta 1.

## Data Availability

The data presented in this study are available on reasonable request from the corresponding author. The data are not publicly available because they contain information that could compromise the privacy of the child participants and because the institutional ethics approval does not cover public data deposition.

## Supplementary Materials

The following supporting information can be downloaded at: https://www.mdpi.com/article/10.3390/children13091242/s1, Table S1: Full specification of the clinical and biomarker-specific multivariable Cox proportional hazards models for sustained treatment escalation over 12 months in the development cohort (Şanlıurfa); Figure S1: Calibration of predicted and observed 12-month risk of sustained treatment escalation; and the completed TRIPOD checklist.
