# Supplementary material for: Biomarkers of Treatment-Escalation Risk by Obesity Status in Pediatric Asthma: A Development and External Validation Cohort Study
Source: Children (Basel). 2026 Sep 14;13(9):1242. doi: 10.3390/children13091242 (PMC13605966; doi:10.3390/children13091242)
Supplement: Supplementary file 1 [file children-13-01242-s001.zip › children-4522189-supplementary.pdf]

children

Supplementary Materials

Biomarkers of Treatment-Escalation Risk by Obesity Status in Pediatric Asthma: A Development and External Validation Cohort Study

Table S1. Full specification of the clinical and biomarker-specific multivariable Cox proportional hazards models for sustained treatment escalation over 12 months in the development cohort (Şanlıurfa).

Model 0. Clinical model

Predictor	β coefficient	HR (95% CI)
Age, per 1-year increase	−0.0162	0.98 (0.87–1.11)
Male sex (vs female)	0.1284	1.14 (0.71–1.82)
Obesity (vs normal weight)	0.2445	1.28 (0.78–2.10)
Baseline FEV_1, per 1% predicted	−0.0154	0.98 (0.96–1.01)
Uncontrolled asthma (vs controlled)	0.4619	1.59 (0.95–2.65)
Baseline GINA step, per 1-step increase	0.2215	1.25 (0.88–1.77)

Model 0 parameter: 12-month baseline cumulative hazard, $H_0(12) = 0.6514$.

Model A. TGF-β1 model

Predictor	β coefficient	HR (95% CI)
Age, per 1-year increase	−0.0198	0.98 (0.87–1.11)
Male sex (vs female)	0.1310	1.14 (0.71–1.83)
Obesity (vs normal weight)	0.2624	1.30 (0.79–2.14)
Baseline FEV_1, per 1% predicted	−0.0154	0.98 (0.96–1.01)
Uncontrolled asthma (vs controlled)	0.4700	1.60 (0.96–2.67)
Baseline GINA step, per 1-step increase	0.2231	1.25 (0.88–1.78)
TGF-β1, per 500 pg/mL	−0.1054	0.90 (0.63–1.30)
TGF-β1 (per 500 pg/mL) × obesity	0.8560	2.35 (1.52–3.64)

Model A parameter: 12-month baseline cumulative hazard, $H_0(12) = 0.6720$.

Model B. sST2 model

Predictor	β coefficient	HR (95% CI)
Age, per 1-year increase	−0.0198	0.98 (0.87–1.11)
Male sex (vs female)	0.1398	1.15 (0.72–1.84)
Obesity (vs normal weight)	0.2469	1.28 (0.77–2.13)
Baseline FEV_1, per 1% predicted	−0.0141	0.99 (0.97–1.01)
Uncontrolled asthma (vs controlled)	0.4824	1.62 (0.97–2.70)
Baseline GINA step, per 1-step increase	0.2311	1.26 (0.89–1.79)
sST2, per 10 ng/mL	−0.3567	0.70 (0.37–1.32)
sST2 (per 10 ng/mL) × obesity	0.7621	2.14 (1.10–4.17)

Model B parameter: 12-month baseline cumulative hazard, $H_0(12) = 0.6952$.

Model C. CXCL8 model

Predictor	β coefficient	HR (95% CI)
Age, per 1-year increase	−0.0182	0.98 (0.87–1.11)
Male sex (vs female)	0.1222	1.13 (0.70–1.81)
Obesity (vs normal weight)	0.3148	1.37 (0.82–2.28)

 https://doi.org/10.3390/children13091242

Baseline FEV_1, per 1% predicted	−0.0162	0.98 (0.96–1.01)
Uncontrolled asthma (vs controlled)	0.4447	1.56 (0.93–2.61)
Baseline GINA step, per 1-step increase	0.2151	1.24 (0.87–1.76)
CXCL8, per 10 pg/mL	0.7227	2.06 (1.67–2.54)
CXCL8 (per 10 pg/mL) × obesity	−0.8055	0.45 (0.30–0.67)

Model C parameter: 12-month baseline cumulative hazard, $H_0(12) = 0.6385$.

Model D. IGF-1 model

Predictor	β coefficient	HR (95% CI)
Age, per 1-year increase	−0.0222	0.98 (0.87–1.11)
Male sex (vs female)	0.1284	1.14 (0.71–1.82)
Obesity (vs normal weight)	0.2852	1.33 (0.80–2.21)
Baseline FEV_1, per 1% predicted	−0.0154	0.98 (0.96–1.01)
Uncontrolled asthma (vs controlled)	0.4600	1.58 (0.95–2.64)
Baseline GINA step, per 1-step increase	0.2200	1.25 (0.88–1.77)
IGF-1, per 50 ng/mL	0.1310	1.14 (0.88–1.47)
IGF-1 (per 50 ng/mL) × obesity	−0.0182	0.98 (0.68–1.41)

Model D parameter: 12-month baseline cumulative hazard, $H_0(12) = 0.6601$.

β coefficients represent log hazard ratios, and HRs were calculated as exp(β). Binary indicators were coded as follows: male sex = 1 and female sex = 0; obesity = 1 and normal weight = 0; and uncontrolled asthma = 1 and controlled asthma = 0. Age was entered in years, baseline FEV_1 as percent predicted, and baseline GINA treatment step as the ordinal numeric step value.

Biomarkers were centered at prespecified medians of the full development cohort (n = 292), including healthy controls, and scaled before model fitting. The centered and scaled variables used for both the biomarker main effect and the biomarker-by-obesity interaction were defined as ZTGF = (TGF-β1 − 1500)/500, ZsST2 = (sST2 − 18)/10, ZCXCL8 = (CXCL8 − 40)/10, and ZIGF-1 = (IGF-1 − 230)/50. TGF-β1 values are serum-equivalent concentrations after correction for the mandatory 1:10 serum/plasma activation dilution described in Section 2.3. The biomarker main-effect coefficient therefore represents the biomarker association per displayed increment in the normal-weight reference group, whereas the corresponding association in participants with obesity is exp(βbiomarker + βinteraction). The obesity main-effect coefficient represents the association of obesity with the outcome at the biomarker reference median, conditional on the other covariates in the model.

For an individual participant, the linear predictor is calculated as LP = ΣβjXj using the coefficients and predictor coding shown for the relevant model. Twelve-month absolute risk is then calculated as $P(T \leq 12 \mid X) = 1 - \exp[-H_0(12) \times \exp(LP)]$. The model-specific $H_0(12)$ values shown above correspond to the displayed predictor coding, scaling, and transformations. The same coefficients, predictor coding, scaling constants, biomarker-centering values, and $H_0(12)$ values were applied to the external validation cohort from Van without refitting or recalibration. Coefficients are displayed to four decimal places; all predictions were calculated using the full-precision model estimates.

Abbreviations: CI, confidence interval; CXCL8, C-X-C motif chemokine ligand 8; FEV_1, forced expiratory volume in 1 second; GINA, Global Initiative for Asthma; $H_0(12)$, 12-month baseline cumulative hazard; HR, hazard ratio; IGF-1, insulin-like growth factor 1; LP, linear predictor; sST2, soluble suppression of tumorigenicity 2; TGF-β1, transforming growth factor beta 1.

https://doi.org/10.3390/children13091242

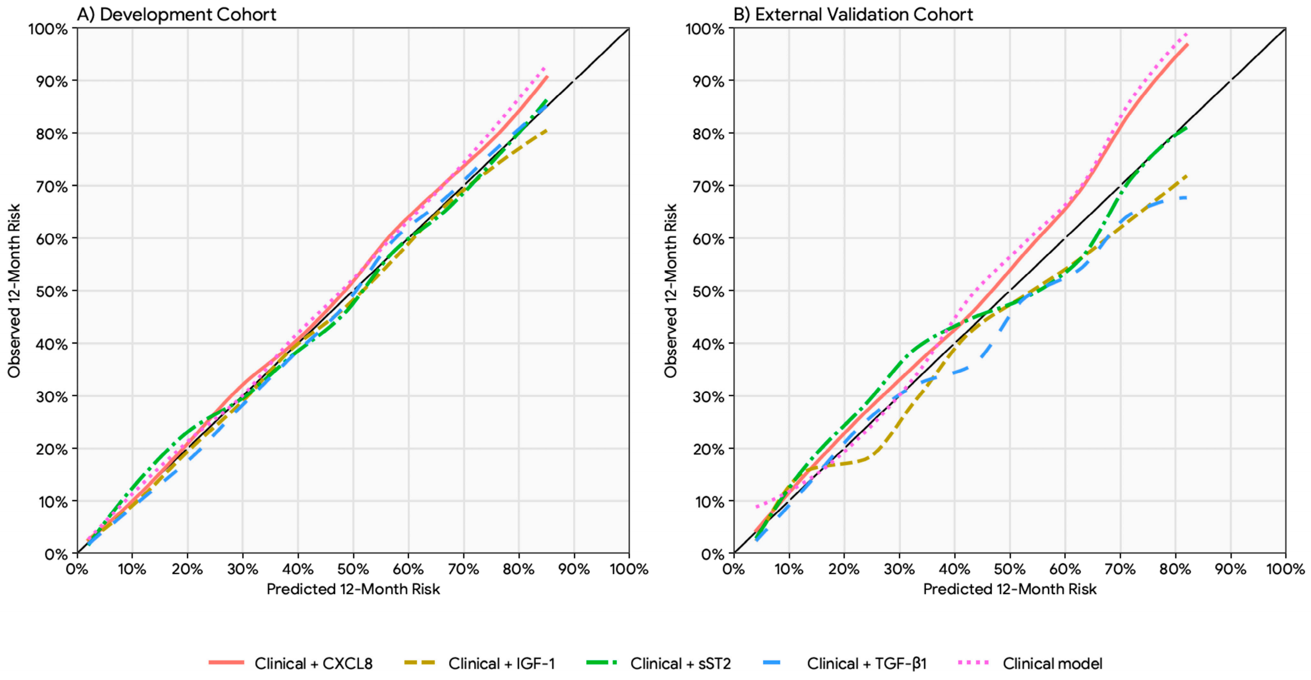

Figure S1. Calibration of predicted and observed 12-month risk of sustained treatment escalation.

Calibration curves are shown for the development cohort from Şanlıurfa (A) and the external validation cohort from Van (B). Observed 12-month risks were estimated using local Kaplan–Meier estimates to account for censoring. The clinical model included age, sex, obesity status, baseline FEV_1 percent predicted, uncontrolled asthma, and baseline GINA treatment step. Each biomarker model added the corresponding continuous biomarker and its biomarker-by-obesity interaction. In the external validation cohort, the development-model coefficients, predictor coding, scaling constants, biomarker-centering values, and model-specific 12-month baseline cumulative hazards were applied without refitting or recalibration. Curves are displayed over the observed range of predicted 12-month risks. The diagonal reference line represents perfect calibration.

Abbreviations: CXCL8, C-X-C motif chemokine ligand 8; FEV_1, forced expiratory volume in 1 second; GINA, Global Initiative for Asthma; IGF-1, insulin-like growth factor 1; sST2, soluble suppression of tumorigenicity 2; TGF-β1, transforming growth factor beta 1.

The completed TRIPOD checklist is provided as a separate supplementary file.
