# Supplementary material for: Biomarkers of Treatment-Escalation Risk by Obesity Status in Pediatric Asthma: A Development and External Validation Cohort Study
Source: Children (Basel). 2026 Sep 14;13(9):1242. doi: 10.3390/children13091242 (PMC13605966; doi:10.3390/children13091242)
Supplement: Supplementary file 1 [file children-13-01242-s001.zip › TRIPOD 2015 Checklist.pdf]

TRIPOD 2015 Checklist

Biomarkers of Treatment-Escalation Risk by Obesity Status in Pediatric Asthma: A Development and External Validation Cohort Study

Development and external validation study

Locations refer to the revised manuscript and supplementary materials.

Section / Topic	Item	Type	Checklist item	Location in manuscript
Title and abstract	1	D;V	Identify the study as developing and/or validating a multivariable prediction model, the target population, and the outcome to be predicted.	Title, p. 1.
Title and abstract	2	D;V	Provide a summary of objectives, study design, setting, participants, sample size, predictors, outcome, statistical analysis, results, and conclusions.	Abstract, p. 2.
Introduction	3a	D;V	Explain the medical context and rationale for developing or validating the model, including reference to existing evidence.	Introduction, p. 3.
Introduction	3b	D;V	Specify the objectives, including whether the study reports model development, validation, or both.	Introduction, p. 3.
Methods: Source of data	4a	D;V	Describe the study design or source of data separately for the development and validation data sets.	Section 2.1, pp. 3-4.
Methods: Source of data	4b	D;V	Specify key study dates, including accrual and follow-up dates.	Section 2.1, pp. 3-4.
Methods: Participants	5a	D;V	Specify the study setting, including the number and location of centers.	Section 2.1, pp. 3-4.
Methods: Participants	5b	D;V	Describe eligibility criteria for participants.	Section 2.1, pp. 3-4.
Methods: Participants	5c	D;V	Give details of treatments received, if relevant.	Sections 2.1–2.2; Tables 1 and 5A.
Methods: Outcome	6a	D;V	Clearly define the predicted outcome, including how and when it was assessed.	Section 2.4.
Methods: Outcome	6b	D;V	Report any actions used to blind outcome assessment.	Section 2.4.
Methods: Predictors	7a	D;V	Clearly define all predictors used in the model, including how and when they were measured.	Sections 2.2–2.3; Section 2.5; Supplementary Table S1.
Methods: Predictors	7b	D;V	Report any actions used to blind predictor assessment to outcome and other clinical data.	Section 2.3.
Methods: Sample size	8	D;V	Explain how the study size was determined.	Section 2.8.
Methods: Missing data	9	D;V	Describe how missing data were handled, including details of imputation.	Sections 2.5–2.7; Table 4B note for the mean-imputation sensitivity analysis.
Methods: Statistical analysis	10a	D	Describe how predictors were handled in the analyses.	Section 2.5; Supplementary Table S1, including exact indicator coding and centered/scaled biomarker definitions.
Methods: Statistical analysis	10b	D	Specify the model type, model-building procedures, predictor selection, and internal validation method.	Sections 2.5–2.6.
Methods: Statistical analysis	10c	V	For validation, describe how predictions were calculated.	Section 2.7; Supplementary Table S1.
Methods: Statistical analysis	10d	D;V	Specify all measures used to assess model performance and compare models.	Sections 2.6–2.7.
Methods: Statistical analysis	10e	V	Describe any model updating after validation, if done.	Not applicable. The development models were applied without refitting or recalibration; Section 2.7.
Methods: Risk groups	11	D;V	Describe how risk groups were created, if done.	Not applicable. No clinical risk groups were created. Biomarker tertiles were used only for visualization; Section 2.5; Figure 2.
Methods: Development vs. validation	12	V	For validation, identify differences from the development data in setting, eligibility criteria, outcome, and predictors.	Section 2.1; Tables 1 and 5A; Discussion.

Section / Topic	Item	Type	Checklist item	Location in manuscript
Results: Participants	13a	D;V	Describe participant flow, outcome counts, and follow-up; a diagram may be used.	Section 3.1 and Figure 1; Section 3.5 and Table 5A.
Results: Participants	13b	D;V	Describe participant characteristics, including missing predictor and outcome data.	Table 1; Table 5A.
Results: Participants	13c	V	For validation, compare important variables with the development data.	Tables 1 and 5A; Discussion.
Results: Model development	14a	D	Specify the number of participants and outcome events in each analysis.	Section 3.1; Tables 3 and 4.
Results: Model development	14b	D	Report unadjusted associations between candidate predictors and outcome, if done.	Not applicable. Candidate-predictor screening was not used. Descriptive biomarker-outcome summaries are shown in Table 2.
Results: Model specification	15a	D	Present the full prediction model so that individual predictions can be calculated.	Supplementary Table S1, including all β coefficients, predictor coding, biomarker transformations, and $H_0(12)$.
Results: Model specification	15b	D	Explain how to use the prediction model.	Supplementary Table S1, including the linear-predictor construction and 12-month absolute-risk equation.
Results: Model performance	16	D;V	Report model performance measures with confidence intervals.	Table 4; Table 5B; Figure 3; Supplementary Figure S1. Discrimination and prediction-error estimates are reported with 95% CIs; calibration is reported using intercept/slope estimates and calibration curves, with 95% CIs for external-validation calibration estimates.
Results: Model updating	17	V	Report results from model updating, if done.	Not applicable. No model updating, refitting, or recalibration was performed in external validation; Sections 2.7 and 3.5.
Discussion: Limitations	18	D;V	Discuss study limitations, including sample size, missing data, and generalizability where relevant.	Discussion.
Discussion: Interpretation	19a	V	For validation, discuss performance relative to the development data and other validation evidence.	Discussion.
Discussion: Interpretation	19b	D;V	Provide an overall interpretation considering the objectives, limitations, similar studies, and other evidence.	Discussion.
Discussion: Implications	20	D;V	Discuss potential clinical use and implications for future research.	Discussion and Conclusions.
Other information: Supplementary information	21	D;V	Provide information on supplementary resources, protocols, calculators, or data sets.	Supplementary Materials; Data Availability Statement; Supplementary Table S1; Supplementary Figure S1; completed TRIPOD checklist.
Other information: Funding	22	D;V	Give the source of funding and the role of funders.	Funding statement.

D = development; V = external validation. Items marked “Not applicable” were not performed or were not part of the study design.
